# Genomic Characterization and Safety Evaluation of *Enterococcus lactis* RB10 Isolated from Goat Feces

**DOI:** 10.3390/antibiotics14060612

**Published:** 2025-06-16

**Authors:** Nattarika Chaichana, Sirikan Suwannasin, Jirasa Boonsan, Thunchanok Yaikhan, Chollachai Klaysubun, Kamonnut Singkhamanan, Monwadee Wonglapsuwan, Rattanaruji Pomwised, Siriwimon Konglue, Rusneeta Chema, Manaschanan Saivaew, Komwit Surachat

**Affiliations:** 1 Department of Biomedical Sciences and Biomedical Engineering, Faculty of Medicine, Prince of Songkla University, Hat Yai 90110, Songkhla, Thailand; cnattari@medicine.psu.ac.th (N.C.); 6710330030@psu.ac.th (S.S.); 6610320008@psu.ac.th (J.B.); ythuncha@medicine.psu.ac.th (T.Y.); chollachai.k@psu.ac.th (C.K.); skamonnu@medicine.psu.ac.th (K.S.); siriwimon3395@gmail.com (S.K.); rusneetaneeta20@gmail.com (R.C.); manas.vaew56@gmail.com (M.S.); 2 Division of Biological Science, Faculty of Science, Prince of Songkla University, Hat Yai 90110, Songkhla, Thailand; monwadee.wo@psu.ac.th (M.W.); rattanaruji.p@psu.ac.th (R.P.)

**Keywords:** *Enterococcus lactis*, genome analysis, safety assessment, pangenome, antibacterial activity

## Abstract

**Background:** The genus *Enterococcus* includes a diverse group of bacteria that are commonly found in the gastrointestinal tracts of humans and animals, as well as in various environmental habitats. **Methods:** In this study, *Enterococcus lactis* RB10, isolated from goat feces, was subjected to comprehensive genomic and functional analysis to assess its safety and potential as a probiotic strain. **Results:** The genome of *E. lactis* RB10, with a size of 2,713,772 bp and a GC content of 38.3%, was assembled using Oxford Nanopore Technologies (ONT). Genome annotation revealed 3375 coding sequences (CDSs) and highlighted key metabolic pathways involved in carbohydrate, protein, and amino acid metabolism. The strain was susceptible to important antibiotics, including ampicillin, chloramphenicol, tetracycline, and vancomycin, but exhibited resistance to aminoglycosides, a common trait in *Enterococcus* species with non-hemolytic activity. Genomic analysis further identified two intrinsic antimicrobial resistance genes (ARGs). The strain also demonstrated antimicrobial activity against *Bacillus cereus* DMST 11098 and *Salmonella* Typhi DMST 22842, indicating pathogen-specific effects. Key genes for adhesion, biofilm formation, and stress tolerance were also identified, suggesting that RB10 could potentially colonize the gut and compete with pathogens. Moreover, the presence of bacteriocin and secondary metabolite biosynthetic gene clusters suggests its potential for further evaluation as a biocontrol agent and gut health promoter. **Conclusions:** However, it is important to note that *E. lactis* RB10 was isolated from goat feces, a source that may harbor both commensal and opportunistic bacteria, and therefore additional safety assessments are necessary. While further validation is needed, *E. lactis* RB10 exhibits promising probiotic properties with low pathogenic risk, supporting its potential use in food and health applications.

## 1. Introduction

The genus *Enterococcus* comprises a diverse group of lactic acid bacteria (LAB) that are commonly found in the gastrointestinal tracts (GIT) of humans and animals, as well as in soil, water, and fermented foods [1,2]. These bacteria are widely recognized for their probiotic potential, which includes supporting gut health, modulating the immune system, and maintaining microbial balance in the intestines [3,4]. The probiotic benefits of *Enterococcus* species have been well documented, particularly their ability to compete with pathogens, enhance digestion, and prevent gastrointestinal disorders. While certain *Enterococcus* species, such as *Enterococcus faecium* and *Enterococcus faecalis*, have been extensively studied for their roles in both health and disease [5,6,7], other species, such as *Enterococcus lactis*, remain relatively understudied. *E. lactis* has recently gained attention as a probiotic strain, primarily due to its ability to survive in the GIT, resistance to bile salts, and production of antimicrobial compounds, such as bacteriocins and lactic acid [8]. These traits allow *E. lactis* to be an attractive candidate for food preservation and gut health promotion. For instance, the *E. lactis* strain JDM1 has been shown to exert anti-pathogenic activity in the GIT without inducing toxicity in cytotoxicity tests [9]. Furthermore, strains like *E. lactis* 10NA and 50NA have demonstrated resistance to bile salts, acid tolerance, and antibacterial activity against *Escherichia coli*, *Salmonella* Typhi, and *Clostridioides difficile*. Genomic analysis of these strains revealed no significant associations with virulence or antimicrobial resistance genes (AMR), indicating a low risk of horizontal gene transfer [10]. Moreover, *E. lactis* YHC20 exhibited additional beneficial properties, such as cholesterol-lowering effects and cell surface hydrophobicity [8], further supporting its potential as a safe and effective probiotic. However, like other *Enterococcus* species, *E. lactis* strains exhibit genetic variability, and the potential presence of AMR and virulence factors (VFs) raises concerns about their safety for probiotic applications [11]. Since the properties of probiotic bacteria are strain specific, individual strains within the same species can display significant differences in functional characteristics, including metabolic profiles, antimicrobial resistance, and pathogenic potential. Therefore, a comprehensive evaluation of safety in each strain is crucial before considering its use as a probiotic.

One of the cornerstone documents in probiotic evaluation is the FAO/WHO, Guidelines for the Evaluation of Probiotics in Food (2002) [12], which defines probiotics as “live microorganisms which, when administered in adequate amounts, confer a health benefit on the host”. These guidelines recommend a stepwise assessment beginning with unambiguous molecular and phenotypic strain identification, followed by a thorough safety evaluation, including antibiotic-susceptibility profiling, absence of hemolytic activity and toxin production, and screening for transferable resistance or virulence factors. They further require in vitro functional assays (adhesion, antimicrobial activity) and, where possible, in vivo or clinical studies to substantiate health claims, as well as documentation of product stability and viability through shelf life and gastrointestinal transit.

Genomic analysis, particularly whole genome sequencing (WGS), is an essential tool for assessing safety risks and understanding the functional traits of probiotic candidates. WGS helps differentiate beneficial Enterococcus strains from those with potential safety concerns by identifying key genes linked to probiotic functions. Beneficial strains typically carry genes related to bacteriocin production, lactic acid synthesis, and immune modulation, while ideally lacking transferable antimicrobial resistance genes (ARGs) and significant virulence factors [13]. In contrast, pathogenic strains often show an abundance of mobile genetic elements (MGEs), ARGs, and virulence determinants, which increase their pathogenic potential [14]. Hence, comparative genomic analysis and WGS help identify genes associated with probiotic functions and assess safety for food or therapeutic use.

Functional annotations, in silico safety assessments, and pan-genome analysis provide a comprehensive evaluation of the bacterial genetic traits. Therefore, this study aims to investigate the genomic characteristics and safety of *E. lactis* RB10, isolated from goat feces, through both genomic analysis and in vitro safety assessments. The results revealed a balance of beneficial traits and genetic elements, which may require further evaluation. These findings underscore the importance of comprehensive safety and functionality assessments before considering the strain’s application in food or therapeutic settings, particularly in light of the regulatory frameworks of the FAO/WHO guidelines for probiotic evaluation.

## 2. Results

### 2.1. Antibiotic Susceptibility of E. lactis RB10

The antibiotic susceptibility of *E. lactis* RB10 was assessed by testing with different commonly used antibiotics. The results show that *E. lactis* RB10 is susceptible to several antibiotics, including ampicillin, chloramphenicol, tetracycline, and vancomycin. In contrast, the strain shows resistance to clindamycin, erythromycin, gentamycin, kanamycin, and streptomycin (Table 1).

### 2.2. Hemolysis Activity

**Figure 1:**
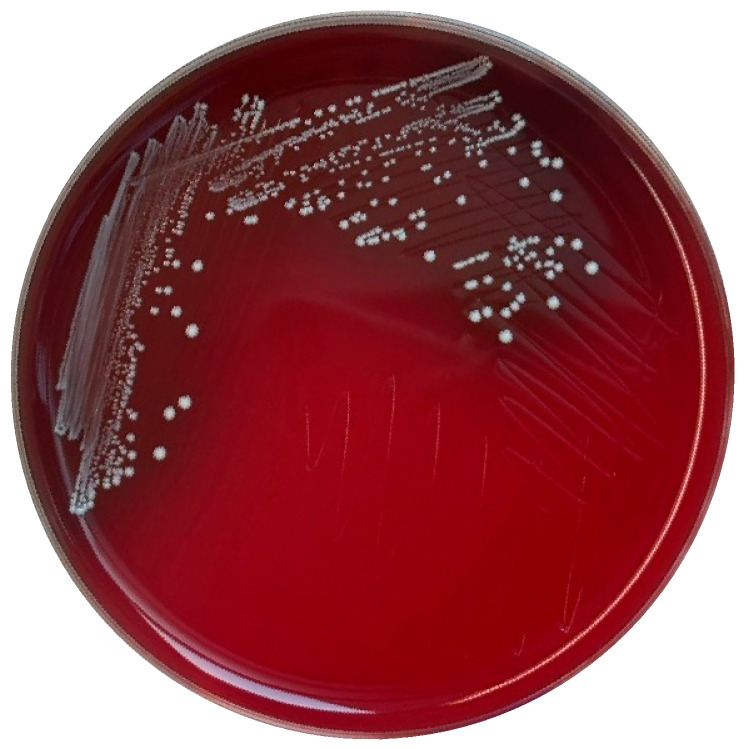
Hemolysis activity of *E. lactis* RB10 on blood agar. The image shows no visible hemolytic zone around the colonies, indicating a γ-hemolysis pattern.

The hemolysis activity of *E. lactis* RB10 was evaluated on the BHI agar containing 5% blood. As shown in Figure 1, the strain showed γ-hemolysis activity with no visible zones of red blood cell lysis around the colonies.

### 2.3. Overview Features of the E. lactis RB10 Genome

WGS analysis of the strain was processed using long-read technology on the ONT platform. The genome assembly resulted in 8 contigs, with an L50 value at the first contig. The total length of the genome is 2,713,772 bp with a GC content of 38.3%. The size and number of the largest contigs of N50 is 1,148,270. The genome contains 3375 CDSs, 66 tRNA genes, 12 rRNA genes, and 1 tmRNA gene. Moreover, 1 repeat region and 233 subsystems are identified. Moreover, the strain was identified as *E. lactis* based on taxonomy analysis using the Genome Taxonomy Database (GTDB). The closest related reference strain is *E. lactis* CCM 8412, with an Average Nucleotide Identity (ANI) value above 98% (Table 2).

**Figure 2:**
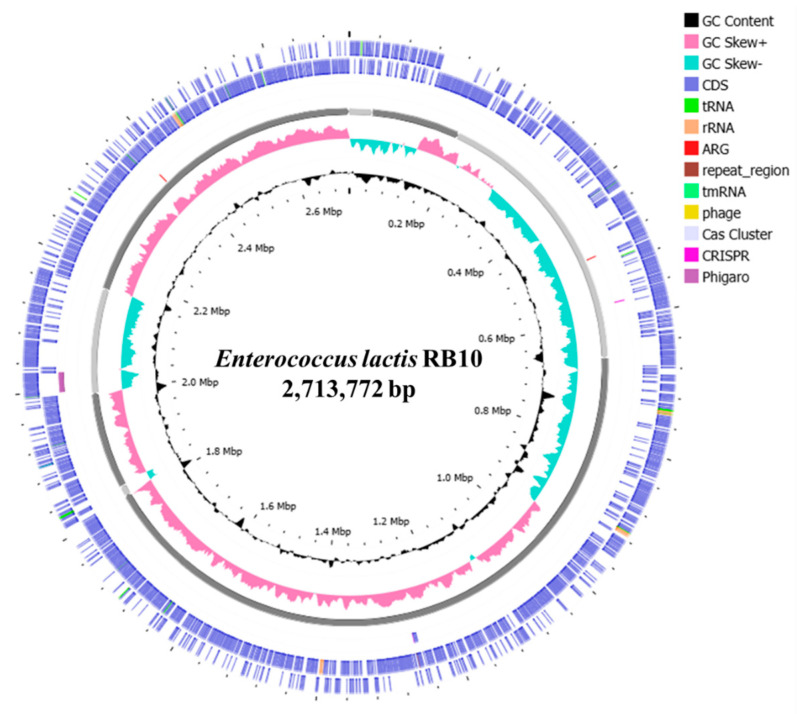
Circular visualization of the *E. lactis* RB10 genome map. Marked information is displayed from the outer circle to the innermost as follows: CDS on the forward strand, CDS on the reverse strand, with tRNA and rRNA, phigaro, CRISPR, ARGs, GC skew, and GC content.

**Figure 3:**
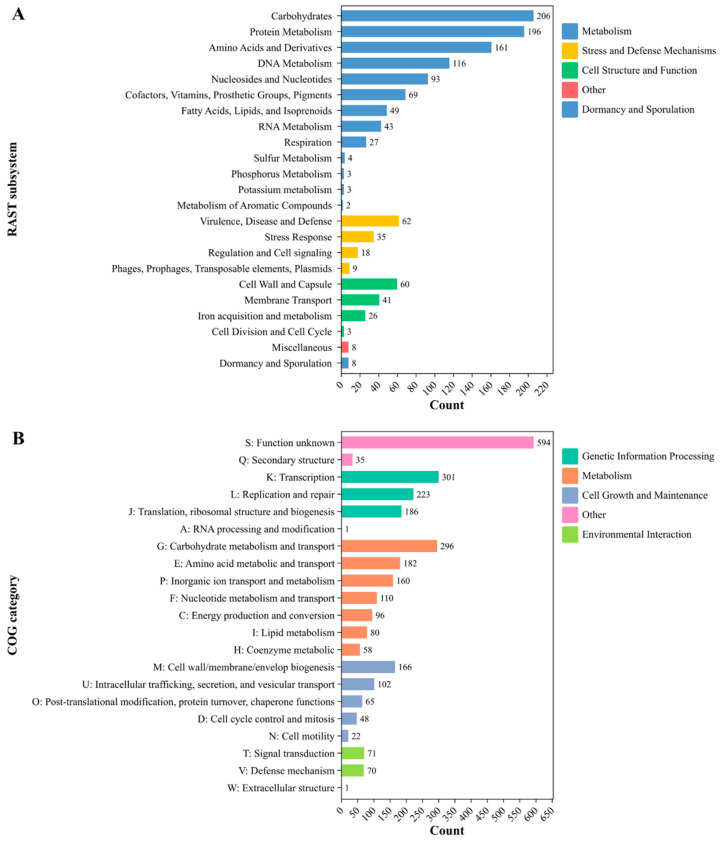
Functional categorization of the *E. lactis* RB10 genome. (**A**) Distribution of the genome into RAST subsystems, with a significant number of genes related to metabolism, stress and defense mechanisms, and cell structure and function. (**B**) Classification of the genome into COG categories, highlighting the predominant roles in genetic information processing, metabolism, cell growth and maintenance, and others.

An overview of the genome features of *E. lactis* RB10 was visualized in a circular genome map illustrated in Figure 2. The circular genome visualizes key genomic elements and various functional elements such as coding sequences (CDS), tRNA, rRNA, and regions associated with mobile genetic elements (MGEs), antimicrobial resistance genes (ARGs), phages, Cas clusters, and CRISPR. In addition, the 233 subsystems of the RAST annotation with 3375 proteins were classified to highlight the most prominent metabolic processes in *E. lactis* RB10, with carbohydrates (206 CDSs), protein metabolism (196 CDSs), and amino acids and derivatives (161 CDSs) representing the most abundant functional categories. Other notable categories include DNA metabolism (116 CDSs), cofactors, vitamins, prosthetic groups, and pigments (69 CDSs), and fatty acids, lipids, and isoprenoids (49 CDSs). In terms of stress and defense mechanisms, virulence, disease, and defense and stress response are the most prominent categories, with 62 and 27 CDSs, respectively (Figure 3A). Moreover, the COG category distribution further categorizes the functional roles of proteins in the strain. The largest proportion of genes is assigned to genetic information processing, specifically transcription (301 CDSs) and replication and repair (223 CDSs). Another substantial group corresponds to metabolism, with carbohydrate metabolism and transport (296 CDSs), amino acid metabolism and transport (182 CDSs), and inorganic ion transport and metabolism (160 CDSs). A significant number of genes are involved in cell growth and maintenance, such as coenzyme metabolism (58 CDSs) and cell wall/membrane/envelope biogenesis (166 CDSs). Other functional categories, including environmental interaction, signal transduction, and defense mechanisms, have moderate representation, while cell motility and extracellular structure have fewer counts (Figure 3B).

### 2.4. Antibiotic Resistance Genes and Virulence Factor Profile of RB10

The safety assessment analysis of *E. lactis* RB10 was performed by identifying ARGs and virulence factor-associated genes through the databases. The results exhibited that the identified AMR genes include *aac(6′)-Ii*, which encodes an aminoglycoside N-acetyltransferase, and *msr(C)*, an ABC-F type ribosomal protection protein conferring macrolide resistance. The virulence factor-related genes identified in *E. lactis* RB10 are more likely to contribute to its probiotic potential rather than pathogenicity. Adhesion genes such as *sgrA*, *scm*, and *acm* are predicted to mediate tight binding to host extracellular matrix components, promoting stable colonization of the intestinal mucosa and competitive exclusion of pathogens. The *ebpC* pilus subunit, together with two sortases (*srtC* variants), likely assembles surface pili that foster biofilm formation. Capsular polysaccharide synthesis genes *(cap8D*, *cpsA/uppS*, and *cpsB/cdsA*) are expected to generate a protective extracellular matrix. Stress response chaperone and protease genes, including *groEL*, *htpB*, *clpC*, *clpE*, and *clpP*, provide protein quality control under thermal, oxidative, and other stresses encountered in environmental stresses (Table S1).

### 2.5. Plasmid and MGE Identification

The identification of the plasmid found that contig 2 of the RB10 assembly harbors two distinct replication initiator genes, each closely related to known lactic acid bacterial plasmids. A repA_N–type replicase (repUS15) shares 98.2% identity over its full length with the repA gene of the *Enterococcus faecium* NRRL B-2354 plasmid pNB2354_1 (CP004064). Co-localized on the same contig, an Inc18-family replicase (repE) exhibits 97.6% identity to the repE gene of the *Lactococcus garvieae* plasmid pKL0018 (AB290882) (Table S2). Moreover, MGE profiling of the *E. lactis* RB10 genome uncovered a remarkably rich mobilome of 257 elements, which partition into five main functional classes. The largest group, replication/recombination/repair, includes core genome maintenance proteins such as RecA, DnaA, TopB, and ParC that safeguard DNA integrity. Integration/excision elements represented by numerous transposases and integrases (e.g., Tra905, TnpA, InsF, XerC/D) suggest frequent site-specific DNA insertions and excisions. Conjugal transfer systems, including Tra operon components and partitioning factors, point to potential plasmid or ICE-mediated gene flow. Stability/defense modules encompass restriction–modification enzymes (HsdR, HsdM) and toxin-antitoxin pairs (RelE), which both stabilize MGEs and protect against foreign DNA. In addition, phage-related genes such as terminases and capsid subunits mark prophage remnants dispersed throughout the assembly (Table S3).

### 2.6. Bacteriocin and Secondary Metabolite-Associated Genes Found in RB10 Genome

**Figure 4:**
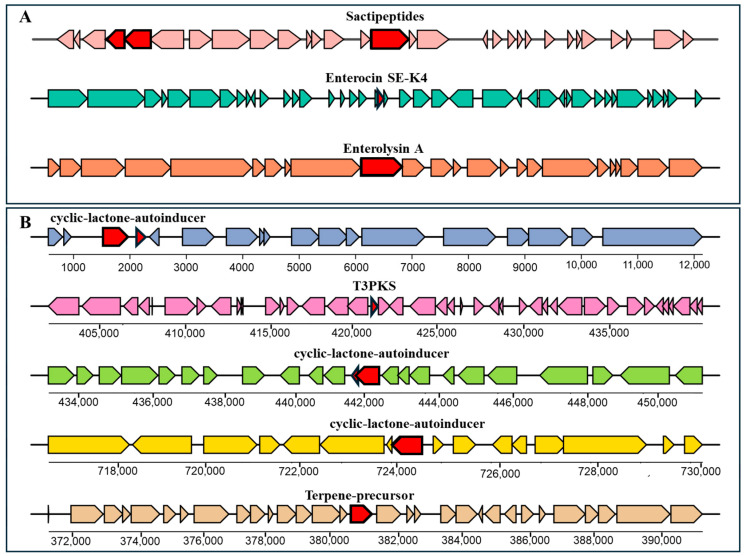
Genome map of *E. lactis* RB10 illustrating annotated bacteriocin biosynthesis gene clusters (**A**) and secondary metabolite biosynthetic gene clusters (**B**). The coordinates on the genome depict the positions of these clusters, with red arrows indicating the location of core biosynthesis genes involved in the production of antimicrobial compounds.

The identification of bacteriocin and secondary metabolite biosynthetic gene clusters (BGCs) in the RB10 genome, conducted using the BAGEL4 and antiSMASH databases, respectively, revealed the presence of multiple biosynthetic clusters associated with antimicrobial activity. The analysis identified three distinct bacteriocin clusters, including sactipeptides from 942,887 to 962,887 bp, enterolysin A between 106,493 and 127,036 bp, and enterocin SE-K4 between 126,029 and 146,143 bp (Figure 4A). In parallel, the secondary metabolite biosynthetic analysis identified a Type III Polyketide Synthase (T3PKS) cluster located from 401,261 to 441,659 bp along with multiple cyclic-lactone-autoinducer clusters, including the first region from 1 to 12,265 bp, the second region from 431,534 to 452,257 bp, and the third region from 713,846 to 734,448 bp (Figure 4B).

### 2.7. Phylogenetic Tree Construction

**Figure 5:**
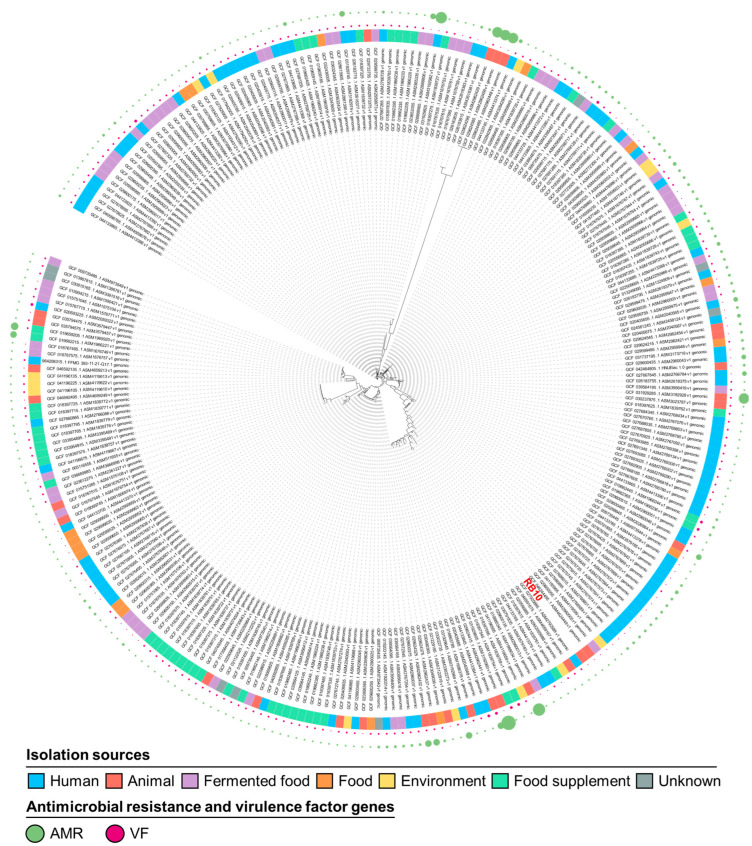
Phylogenetic tree of available *E. lactis* genomes in the NCBI database based on pan-genome SNP analysis. The inner ring represents isolation sources (human, animal, fermented food, food, environment, food supplement, and unknown). The outer rings indicate the presence of virulence factor (VF)-associated genes (purple) and antimicrobial resistance (AMR) genes (green) among strains. RB10 is highlighted in red, showing its genetic relationship with other strains.

The phylogenetic analysis of the available 272 *E. lactis* genomes, based on single-nucleotide polymorphism (SNP) variations from the pan-genome, reveals distinct clustering patterns that differentiate probiotic candidates from potential opportunistic strains (Figure 5). The tree demonstrates that fermented food and food supplement-derived strains form separate clusters from human- and animal-associated isolates. The closest strains to *E. lactis* RB10 based on the phylogenetic tree are 50NA (GCF_027925885.1), TATVAM-FAB81 (GCF_046591675.1), E16 (GCF_020556885.1), and 32-1 (GCF_018397615.1). Among these, 50NA, TATVAM-FAB81, and E16 were isolated from humans, animals, and the environment, respectively, while 32-1 originated from a food supplement. Strains 50NA, TATVAM-FAB81, and E16 harbor a higher number of virulence factors compared to RB10, whereas all four strains share a similar number of ARGs. Moreover, strain 32-1, isolated from a food supplement, exhibited a similar number of both virulence factors and ARGs as RB10.

### 2.8. Comparative Genomic Analysis

**Figure 6:**
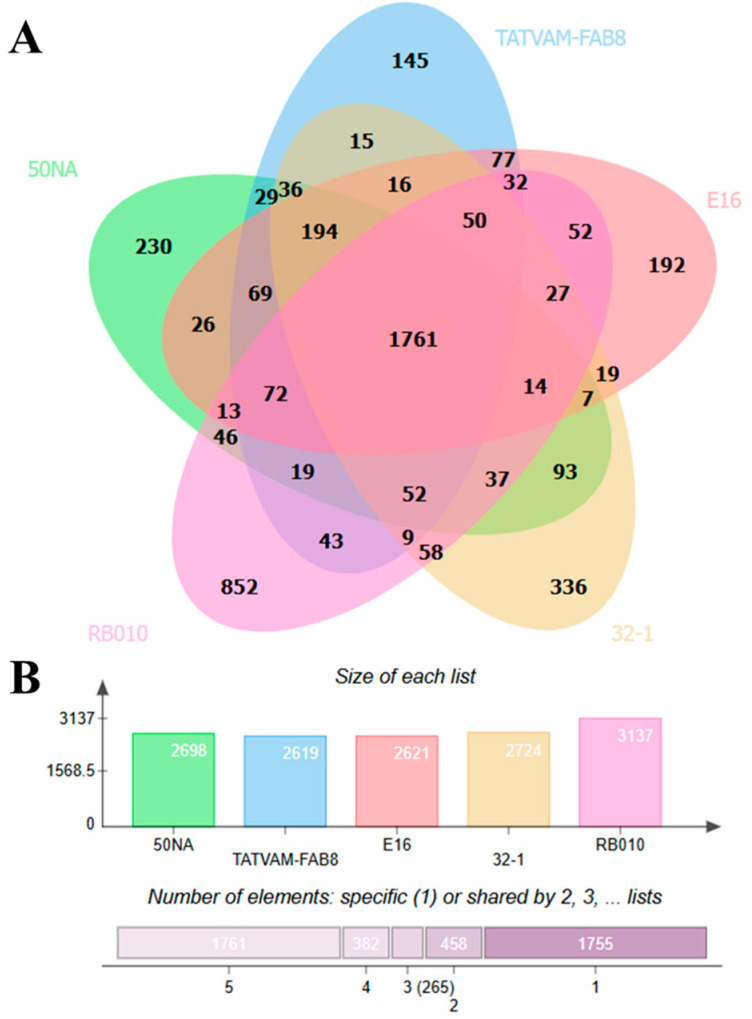
Venn diagram showing the shared and unique genes among *E. lactis* RB10 (pink) and its four closely related strains: 50NA (green), TATVAM-FAB8 (blue), E16 (red), and 32-1 (yellow) (**A**). The diagram shows the distribution of core and strain-specific genes. The bar plot (**B**) represents the total gene count for each strain, with RB10 exhibiting the highest number of genes (3137 total genes), highlighting its genomic diversity compared to the other strains.

The comparative genomic analysis of *E. lactis* RB10 with its closely related strains (50NA, TATVAM-FAB8, E16, and 32-1) reveals insights into core and unique genes among these genomes. The Venn diagram illustrates the distribution of shared and strain-specific genes by showing 1761 core genes that are common among all five genomes, representing the essential genetic backbone of these *E. lactis* strains (Figure 6A). Moreover, RB10 exhibits 3137 total genes, which is the highest among the compared strains. Other strains exhibit a consistent total gene count, with 2698 genes in 50NA, 2619 genes in TATVAM-FAB8, 2621 genes in E16, and 2724 genes in 32-1 (Figure 6B). The RB10 contains 852 unique genes that distinguish it from others. Only 58 genes contribute to their functional diversity and environmental adaptability. Notably, genes such as *merA* (mercuric reductase) and *merR* (mercuric resistance operon regulatory protein) confer mercury resistance, while the presence of *htpB* encodes a heat shock protein. The *scm* gene regulates a collagen adhesin protein, which could enhance the adhesion of the strain to host tissues. Genes such as *clpC* (endopeptidase) and *recN* (DNA repair protein) are important in protein quality control and genomic stability, respectively (Table S4).

### 2.9. Antibacterial Ability Against Foodborne Pathogens

The antibacterial activity of *E. lactis* RB10 was tested against several foodborne pathogens to assess its potential for inhibiting pathogenic bacteria. The results show that *E. lactis* RB10 exhibited significant antimicrobial effects against *B. cereus* DMST 11098 and *S.* Typhi DMST 22842, with inhibition zones of 12.33 ± 0.94 mm and 14.58 ± 0.12 mm, respectively. However, the strain showed no inhibition against *E. coli* O157:H7, *E. faecalis* DMST 4736, and *L. monocytogenes* DMST 17303 (Table 3).

### 2.10. Auto- and Co-Aggregation

**Figure 7:**
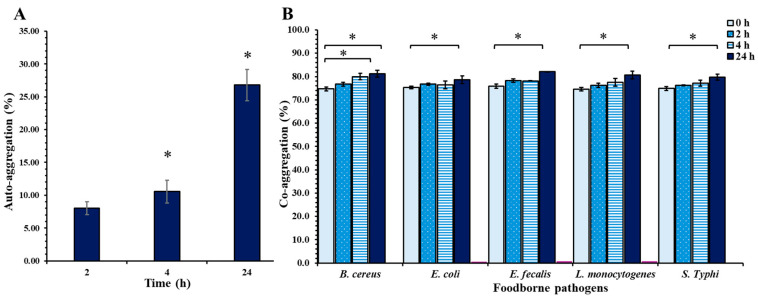
Percentage of auto- and co-aggregation ability of RB10 at each time point. (**A**) The auto-aggregation capacity of RB10 measured at 2, 4, and 24 h. (**B**) Co-aggregation of RB10 with foodborne pathogens assessed at 0, 2, 4, and 24 h. Data represents the mean ± standard deviation (SD) of triplicate experiments. Asterisks (*) indicate statistically significant differences between time points (*p* < 0.05).

The auto- and co-aggregation demonstrate the ability of RB10 to adhere and interact with bacterial cells. The auto-aggregation capacity of RB10, as shown in Figure 7A, was evaluated at 0, 2, 4, and 24 h. The results revealed a significant increase in auto-aggregation over time, with the highest level observed at 24 h (26.79%), compared to 2 h (8.05%) and 4 h (10.57%). The co-aggregation ability of RB10 with foodborne pathogens was assessed at 0, 2, 4, and 24 h. The co-aggregation results demonstrated a marked increase in co-aggregation with specific pathogens at 24 h compared to 0 h. Co-aggregation of *E. lactis* RB10 increased from 74.71% to 81.19% with *B. cereus* DMST 11098 and from 75.26% to 78.62% with *E. coli* O157:H7. Likewise, co-aggregation with *E. faecalis* DMST 4736, *L. monocytogenes* DMST 17303, and *S.* Typhi DMST 22842 rose from 75.86% to 82.05%, 74.59% to 80.58%, and 74.87% to 79.75%, respectively. The highest co-aggregation was observed with *E. faecalis* DMST 4736, followed closely by *B. cereus* DMST 11098 and *L. monocytogenes* DMST 17303. Notably, the co-aggregation trends with all pathogens exhibited a consistent upward trajectory as time progressed (Figure 7B).

## 3. Discussion

*Enterococcus lactis* has recently gained interest in its potential health benefits as a probiotic strain, particularly in promoting gut health and enhancing immune function. *E. lactis* has been found to contribute to intestinal microbiota balance, potentially reducing the risk of gastrointestinal disorders and enhancing digestive health [9,15,16]. Moreover, this strain stands out among *Enterococcus* species due to its absence of virulence and antimicrobial resistance genes, making it an appealing candidate for further study. Safety risks are a primary concern for any bacteria intended for food use, and a few studies have addressed the potential for antibiotic resistance spread in *E. lactis*. While genomic approaches are essential for identifying potential risk factors in probiotic strains, the WGS analysis of *E. lactis* RB10, conducted using long-read sequencing with Oxford Nanopore Technology (ONT), provides a comprehensive genomic characterization, supporting its potential as a safe and beneficial bacterial candidate. The genome consists of 2.71 Mb with a GC content of 38.3%, which falls within the range observed for other *E. lactis* isolates, which range from 2.61 to 2.88 Mb, with GC content varying between 38.00% and 38.33% [17]. This consistency in genome size and GC content across isolates further confirms the genomic coherence of *E. lactis* as a species and suggests that RB10 exhibits typical genomic features for this group of bacteria. The genome annotation revealed an extensive complement of genes underpinning functional diversity and environmental adaptability of RB10, including metabolic versatility and sophisticated regulatory circuits, which collectively facilitate its persistence across a range of ecological niches. A total of 233 subsystems, classified into six major functional groups, suggests a well-structured genome with metabolic flexibility, which is the dominant subsystem category, including carbohydrate metabolism (206 genes), protein metabolism (196 genes), and amino acid metabolism (161 genes). These results highlight its ability to metabolize diverse nutrients, a key feature for gut colonization and survival in fermented food environments. These findings are in line with previous research on *E. lactis*, where carbohydrate metabolism genes have been found to contribute to gut fitness and probiotic traits [10]. In addition, circular genome visualization revealed key genetic elements, including ARGs, CRISPR-Cas systems, and phage-associated regions, which highlight the genetic adaptability of the strain. Previous studies on *E. lactis* as a probiotic strain have reported no concerning pathogenic or transferable antimicrobial resistance genes, further supporting their potential safety for food applications [10]. Moreover, the pathogenic probability score of RB10 (40%) indicates a lower risk compared to known pathogenic *Enterococcus* species, such as *E. avium* and *E. faecalis*, which have been associated with hospital-acquired infections and typically exhibit probability scores exceeding 80% [18,19]. The antibiotic susceptibility of *E. lactis* is crucial for understanding its safety and efficacy as a probiotic, as it determines the ability of the strain to survive in the presence of various antibiotics commonly used in clinical settings. In this study, the antibiotic susceptibility profile of *E. lactis* RB10 indicates that the strain is susceptible to key clinically important antibiotics, including ampicillin, chloramphenicol, tetracycline, and vancomycin, which are widely used in medical treatments. However, resistance to clindamycin, erythromycin, gentamycin, kanamycin, and streptomycin suggests the presence of intrinsic or acquired antimicrobial resistance mechanisms, a characteristic commonly observed in *Enterococcus* species [20,21]. Resistance to clindamycin and erythromycin in RB10 is consistent with the presence of the chromosomal *msrC* efflux gene, while aminoglycoside resistance (gentamicin, kanamycin, streptomycin) likely occurs from intrinsic acetyltransferases (e.g., *aac(6′)-Ii*) and native efflux pumps. The aminoglycoside resistance pattern in RB10 aligns with findings from previous studies, where *E. lactis* strains isolated from human fecal samples [10] and other environments [17] also exhibited high resistance to aminoglycosides. These findings indicate that aminoglycoside resistance may be an intrinsic characteristic of *E. lactis* rather than a result of horizontal gene acquisition. The genomic analysis of *E. lactis* RB10 provides additional insights into its AMR profile. Using the CARD database, only two AMR genes were identified, including *msrC* and *aac(6′)-Ii*, both of which are well known to be chromosomally encoded and intrinsic to *Enterococcus* species. The *msrC* encodes an ABC-F ribosomal protection protein that confers efflux pump associated with macrolide resistance (e.g., erythromycin), while *aac(6′)-Ii* encodes an aminoglycoside-modifying acetyltransferase. In RB10, neither gene is flanked by transposase, integrase, or conjugation machinery, consistent with numerous surveys showing that *aac(6′)-Ii* and *msrC* rarely mobilize under standard conditions [20]. The presence of only these two genes in the genome suggests that RB10 does not harbor a high number of AMR determinants, which reinforces its low-risk profile for horizontal transfer. Phenotypically, disk-diffusion assay of AMR profile of RB10 revealed resistance to multiple aminoglycosides despite the presence of only *aac(6′)-Ii*. One explanation is that other chromosomal factors, such as efflux pumps or ribosomal mutations, may contribute to elevated aminoglycosides without adding new AMR genes. For example, intrinsic efflux systems (e.g., EfrAB) are common in *Enterococcus* and can modestly increase the aminoglycoside concentration required for inhibition [22]. Thus, the discordance between genotype and phenotype likely reflects such intrinsic, non-mobile resistance mechanisms rather than the acquisition of novel AMR cassettes. Moreover, the genetic neighborhood around both *msrC* and *aac(6′)-Ii* was also investigated and found no evidence of nearby insertion sequences, integrative and conjugative elements, or small mobilizable plasmids. This is in line with observations from other *E. lactis* and *E. faecium* genomes, where *aac(6′)-Ii* and *msrC* are stably maintained on the chromosome [10,21]. Overall, these findings indicate that the resistome of *E. lactis* RB10 is restricted to intrinsic, nontransferable resistance markers, with no evidence of acquired or MGE-associated AMR genes. Combined with its susceptibility to key clinical antibiotics, this profile suggests a low risk of horizontal gene transfer and aligns with EFSA guidelines for probiotic safety assessment. Another important safety criterion for probiotic candidates is hemolytic activity. The results showed that *E. lactis* RB10 exhibited no hemolysis on BHI agar supplemented with 5% sheep blood, confirming γ-hemolysis. Because sheep erythrocytes are highly sensitive to enterococcal hemolysins, this result reliably indicates the absence of hemolytic activity and supports its safety for food applications [23]. The absence of hemolysis is a key criterion in probiotic selection, as it ensures the strain does not induce red blood cell lysis or pose a risk of host tissue damage, reinforcing its potential suitability for human consumption. Similar findings have been reported in other *E. lactis* strains, where gamma-hemolytic isolates were considered safe for probiotic use [10,16]. In addition, the virulence factor-related genes presented in *E. lactis* RB10, such as adhesion-related proteins (*sgrA*, *scm*, *acm*), are more likely to support its probiotic characteristics than pathogenicity. These genes enable the strain to adhere to the intestinal mucosa, establish a stable presence, and compete with pathogens in the gut [24,25,26]. The biofilm formation gene (*ebpC*) further enhances its ability to form protective biofilms, which aid in resisting host immune responses and digestive enzymes [27]. While capsule synthesis genes (*cap8D* and *cpsA*) are typically associated with virulence in pathogens, they can also be beneficial for probiotics by improving resilience against stress conditions [28]. Moreover, other stress response genes such as *htpB* (heat shock protein) and *groEL* (chaperonin) ensure protein stability under stress, further supporting the viability of this strain in fluctuating conditions such as temperature changes and acidic pH in the stomach [29,30]. The discovery of a plasmid backbone in *E. lactis* RB10 harboring both a repA_N–type (repUS15) and an Inc18-family replicase (rep1) located on the same contig. The detection of an Inc18-type replication module implies that RB10 may inherently be capable of acquiring and disseminating accessory genes under selective pressure [31]. Although our in silico screen did not reveal any such cargo, full plasmid closure and subsequent functional assays are warranted to exclude hidden transfer capabilities and to confirm that RB10 does not serve as a reservoir for unwanted traits. More broadly, the extensive mobilome mapped across the RB10 genome, including hundreds of elements dedicated to replication/recombination/repair, integration/excision, conjugal transfer, stability/defense, and phage functions, highlights a highly dynamic genomic landscape. While many of these elements (e.g., RecA, DnaA, restriction–modification systems) are integral to chromosome maintenance and defense, the abundance of transposases, integrases, and transfer machinery shines a capacity for genome plasticity [32,33,34]. From a safety standpoint, this genetic fluidity necessitates rigorous phenotypic and molecular monitoring, particularly under conditions that might induce MGE activation, to confirm that RB10 remains genetically stable and free of emergent resistance or virulence determinants during industrial or clinical use. The genomic analysis of *E. lactis* RB10 revealed the presence of several bacteriocin and secondary metabolite BGCs, indicating its considerable antimicrobial potential, a key trait for probiotics aimed at inhibiting pathogenic bacteria in the gastrointestinal tract. In silico screening identified sactipeptides, enterolysin A, and enterocin SE-K4, which are known for their antimicrobial properties. These bacteriocins, commonly produced by *Enterococcus* species, are effective against Gram-positive bacteria, helping the strain compete with harmful microbes [35,36]. Sactipeptides found in *Enterococcus* species are antimicrobial peptides cross-linked by sulfur-to-alpha carbon thioether bonds that belong to the RiPPs (ribosomally synthesized and post-translationally modified peptides) class that demonstrates a range of biological activities, including antibacterial effects [37]. This peptide class has been identified in the *Enterococcus mundtii* 203 genome [38]. Enterolysin A works by binding to the bacterial cell wall, hydrolyzing the peptidoglycan layer, leading to cell lysis and bacterial death [39]. Enterocin SE-K4, on the other hand, targets the bacterial cell membrane, forming pores that cause leakage of cellular contents and result in cell death [13]. These bacteriocins have also been identified in other *Enterococcus* species, including those isolated from wild marine animals in Southern Brazil [40] and from Cameroonian infants [41], further emphasizing their antimicrobial properties across different environments. In parallel, secondary metabolite biosynthetic analysis revealed a T3PKS cluster, associated with the production of polyketide metabolites, which are often antimicrobial and have anticancer properties [42]. Moreover, the identification of cyclic-lactone-autoinducer clusters suggests that *E. lactis* RB10 may produce compounds involved in quorum sensing and interbacterial communication [43]. These compounds help regulate bacterial behavior, including biofilm formation, virulence factor expression, and the production of antimicrobial compounds. This ability to modulate gut microbiota and compete with pathogens supports the probiotic potential of the strain [44]. The identification of these BGCs is consistent with findings from previous studies on *Enterococcus* strains isolated from Cameroonian infants, further supporting the antimicrobial potential of *Enterococcus* species in various environments [41]. However, these predictions have not yet been confirmed for the active compounds, which will therefore constitute the focus of future work.

The phylogenetic analysis of *E. lactis* RB10, conducted using SNP variations from the pan-genome of 272 available *E. lactis* genomes, provides valuable insights into the genetic relationships and functional potential of this strain. The phylogenetic tree reveals distinct clustering patterns, differentiating probiotic candidates from potential opportunistic strains. Notably, strains derived from fermented food and food supplements cluster separately from those isolated from humans and animals, indicating that the environmental origin of the strain influences its genomic characteristics and potential functionality. The closest strains to RB10 based on the phylogenetic tree are 50NA, TATVAM-FAB81, E16, and 32-1. The strains 50NA, TATVAM-FAB81, and E16 were isolated from humans, animals, and the environment, respectively, while 32-1 originated from a food supplement. Comparative genomic analysis reveals that 50NA, TATVAM-FAB81, and E16 harbor more virulence factors than RB10, highlighting the potential pathogenicity of these strains. However, all four strains share a similar antimicrobial resistance gene (ARG) profile, suggesting that the resistance of RB10 is comparable to other closely related strains. While the presence of virulence-related genes in these strains suggests that they may have a higher pathogenic potential, the similar ARG counts suggest that the resistance profile of RB10 is consistent with that of other closely related strains. Interestingly, 32-1, which was isolated from a food supplement, exhibited similar virulence factors and ARG counts to RB10, indicating a comparable safety profile in terms of resistance and pathogenicity. Despite the proximity of RB10 to strains with more virulence factors, the absence of mobile genetic elements (MGEs) and the relatively low number of virulence factors in RB10 reduce the likelihood of horizontal gene transfer or enhanced pathogenicity. The results suggest that while the phylogenetic proximity to more virulent strains warrants attention, the genetic profile of RB10 appears favorable for probiotic use, with no significant evidence of acquired virulence genes or high-risk ARGs. However, in vivo studies and functional validation are still crucial to confirm the safety and probiotic efficacy of RB10 in clinical applications. The Venn diagram illustrates the distribution of core and unique genes among the five strains, showing that 1761 core genes are shared by all, representing the essential genetic backbone of these *E. lactis* strains. Interestingly, RB10 contains 3137 total genes, which is the highest gene count among the compared strains, suggesting a genetically larger genome compared to the others. Out of the 852 unique genes found in RB10, some are crucial for functional diversity and environmental adaptability. For example, *merA* and *merR*, associated with mercury resistance, provide the strain with the ability to survive in toxic environments contaminated with mercury [45]. In addition, the presence of *htpB*, a heat shock protein, suggests that RB10 is well equipped to survive in the harsh conditions [46]. The *scm* gene, encoding a collagen adhesin protein, enhances the adhesion ability of the strain to host tissues, a critical trait for intestinal colonization, which is important for probiotics [25]. Genes such as *clpC* (endopeptidase) and *recN* (DNA repair protein) are essential for protein quality control and genomic stability, respectively, ensuring that RB10 maintains resilience under stress and genomic integrity, key factors for its long-term survival in the gastrointestinal environment [47,48]. While RB10 exhibits several functional traits associated with probiotic potential, 794 genes (93.2%) remain of unknown function, requiring further studies to fully characterize their role in bacterial physiology and function or uncover novel stress-response systems, secretion pathways, or antimicrobial enzymes in the *E. lactis* RB10. This result might represent a rich source of unexplored probiotic and adaptive functions. Overall, the comparative genomic analysis of *E. lactis* RB10 with closely related strains highlights its probiotic potential and functional versatility. All in silico analysis in RB10 remains purely predictive, lacking other assays to confirm gene presence, transcriptional activity, and true functional roles, which is the limitation of this study.

To evaluate the antimicrobial potential of *E. lactis* RB10, its antibacterial activity was tested against several foodborne pathogens to determine its efficacy in inhibiting the growth of harmful microorganisms. The results highlight the pathogen-specific antimicrobial activity of *E. lactis* RB10, underscoring its potential as a biocontrol agent or probiotic strain. The strain exhibited significant inhibition against *B. cereus* DMST 11098 and *S.* Typhi DMST 22842, suggesting that its antimicrobial effects are selective. This narrow spectrum of antagonism is likely attributable to the specific mode of action of the predicted antibacterial compounds in RB10, which are effective only against certain bacteria under the tested conditions. Factors such as the specific target structures of these compounds, differences in cell envelope composition, or suboptimal induction of antimicrobial production may explain the lack of activity against some pathogenic bacteria. These findings are consistent with genomic analysis, where the presence of metabolites could explain its ability to target specific pathogens effectively. Previous studies on the antibacterial activity of *E. lactis* have shown variable effects, with each strain demonstrating different levels of efficacy against specific pathogens [49,50]. Additional properties for competing with pathogens, the auto-aggregation and co-aggregation abilities of *E. lactis* RB10, highlight its potential for gut colonization and pathogen competition, key traits for probiotic strains. Auto-aggregation results showed a significant increase over time, with the highest aggregation observed at 24 h compared to the initial time points. This result suggests that *E. lactis* RB10 has a moderate self-adhesion capability, which is essential for establishing a stable presence in the gastrointestinal tract, where bacterial cells need to adhere to mucosal surfaces for long-term survival. The co-aggregation ability was assessed by measuring the interaction between RB10 and several foodborne pathogens at different time points. The results showed a marked increase in co-aggregation with *E. faecalis* DMST 4736, *B. cereus* DMST 11098, and *L. monocytogenes* DMST 17303 at 24 h. The consistent increase in co-aggregation over time indicates that *E. lactis* RB10 has the ability to adhere to and interact with harmful pathogens, which is crucial for competitive exclusion in the gut, helping prevent pathogen colonization and promoting a healthy gut microbiome. The previous study of the auto-aggregation ability of *E. lactis* PMD74 was shown to be 41 ± 1.00%, while co-aggregation with pathogens showed an ability with *S. enterica* serotype Typhimurium SL1344, *S. aureus* ATCC 6538, and *E. coli* ATCC 26922 [51]. These findings suggest that *E. lactis* RB10 not only has strong auto-aggregation ability for gut colonization but also a significant capacity for co-aggregation with foodborne pathogens, supporting its probiotic potential in competing with harmful microorganisms in the gastrointestinal tract. Although goat feces can be a useful source of novel probiotic candidates, their environmental origin also carries potential hazards that must be carefully managed. Fecal isolates may co-harbor undetected zoonotic pathogens, enteric viruses, or antimicrobial-resistant bacteria acquired in the animal gut or through environmental exposure. Moreover, farm environments can introduce heavy metals, pesticides, and other contaminants that might persist in microbial populations. To mitigate these risks, *E. lactis* RB10 must therefore undergo rigorous phenotypic evaluation, including pathogen challenge assays, hemolysis testing in sensitive erythrocytes, and in vivo trials in healthy, immunocompetent, and immunocompromised models, to confirm that it cannot translocate or cause opportunistic infections under dysbiotic conditions. Only after meeting the FAO/WHO guidelines for probiotic safety should goat-feces-derived strains be advanced toward food-grade applications. Indeed, the genomic information of *E. lactis* RB10 provides valuable insights into the mechanisms by which bacteria adapt to specific environments. It also reveals the genetic functions of its beneficial capabilities, including antimicrobial activities and safety features, contributing to a better understanding of its potential strain.

## 4. Materials and Methods

### 4.1. Bacterial Strains and Culture Conditions

*Enterococcus lactis* RB10 was isolated from goat feces in a local area of Hatyai, Songkhla, Thailand. The bacterial isolate was cultured on de Man, Rogosa, and Sharpe (MRS) agar (Himedia, Mumbai, India) and incubated at 37 °C for 48 h. Foodborne pathogens, including *Bacillus cereus* DMST 11098, *Escherichia coli* O157:H7, *Enterococcus faecalis* DMST 4736, *Listeria monocytogenes* DMST 17303, and *Salmonella* Typhi DMST 22842, were cultured in tryptic soy broth (TSB) (Himedia, Mumbai, India) at 37 °C for 24 h.

### 4.2. Antimicrobial Susceptibility Test

The antimicrobial susceptibility testing (AST) of *E. lactis* RB10 was carried out following the Kirby-Bauer disk diffusion method using Brain Heart Infusion (BHI) agar (Himedia, Mumbai, India) with the *E. lactis* RB10 inoculum of approximately 10^8^ CFU/mL [52]. The following antibiotic disks (Himedia, Mumbai, India) were used including ampicillin (10 μg), chloramphenicol (30 μg), clindamycin (2 μg), erythromycin (10 μg), gentamicin (10 μg), streptomycin (10 μg), tetracycline (30 μg), and vancomycin (30 μg). The antibiotic disks were placed on the inoculated plates and then incubated at 37 °C for 16–18 h. The diameter zone of inhibition values was measured and interpreted according to the cut-off points given by the Clinical and Laboratory Standards Institute (CLSI) guidelines. Moreover, *S. aureus* ATCC 25923 was used as a positive control strain, while sterile water was applied as a negative control to ensure the accuracy of the AST results. The experiment was conducted in three biological replicates.

### 4.3. Hemolysis Assay

Hemolytic activity was evaluated using blood agar plates as previously described. *E. lactis* RB10 was streaked onto BHI agar plates supplemented with 5% blood and incubated at 37 °C for 48 h. The hemolytic patterns were observed and classified as follows: α-hemolysis (partial hemolysis, indicated by a greenish discoloration around the colonies), β-hemolysis (complete hemolysis, shown by a clear zone surrounding the colonies), and γ-hemolysis (no hemolysis, with no visible zone around the colonies). *Staphylococcus aureus* ATCC 25923 was used as the positive control for β-hemolysis activity, while sterile water was used as a negative control to validate the assay [9]. The test was performed in three biological replicates.

### 4.4. DNA Extraction and Whole-Genome Sequencing

The total genomic DNA (gDNA) of *E. lactis* RB10 was extracted using the ZymoBIOMICS DNA Miniprep Kit (Zymo Research, Irvine, CA, USA), following the manufacturer’s instructions. The quantity and quality of the extracted gDNA were evaluated using a Qubit^®^ Fluorometer (Invitrogen, Carlsbad, CA, USA), and DNA integrity was verified through agarose gel electrophoresis. The purified gDNA was then used for long-read sequencing on the Oxford Nanopore Technologies (ONT) platform. The gDNA library was prepared with the Rapid Barcoding Kit 24 V14 (SQK-RAK114.24, Oxford Nanopore Technologies, Oxford, UK) as per the manufacturer’s guidelines. The prepared gDNA library was loaded onto an R10 flow cell and sequenced using the MinION Mk1C device (Oxford Nanopore Technologies, Oxford, UK) following the provided protocol.

### 4.5. Genome Assembly and Functional Annotation

The long-read sequences were processed using the automated pipeline Bactopia v3.0.1 for comprehensive sequence analysis [53]. The genome was assembled with Flye v2.9.5 [54] and annotated via Prokka v1.14.6 [55]. The bacterial species was identified using the Genome Taxonomy Database (GTDB). Subsequently, the draft genome was visualized using the Proksee web server (https://proksee.ca/, accessed on 16 May 2024), providing a detailed circular representation of genomic features. Functional annotations were achieved using Rapid Annotation using Subsystem Technology (RAST) [56] and the eggNOG-mapper v2.1.12 [57] for the classification of predicted proteins into clusters of orthologous groups (COGs) [58]. Moreover, prophage regions were predicted with Phigaro v2.4.0 [59], while regions containing clustered regularly interspaced short palindromic repeats (CRISPR) were identified using the CRISPRFinder server [60].

### 4.6. In Silico Safety Assessment

The safety assessment of the bacterial genome involved predicting genes associated with potential risks, such as pathogenicity, virulence factors, and antimicrobial resistance (AMR). Pathogenic genes were identified through BLASTP v2.8.1+, while virulence factor-associated genes were predicted with the Virulence Factor Database (VFDB). The AMR genes were identified using ResFinder v4.1 [61] and RGI v6.0.0 through the Comprehensive Antibiotic Resistance Database (CARD) [62]. Moreover, the presence of plasmid was mapped by PlasmidFinder v2.0 [63], while mobile genetic elements (MGEs) were identified using the mobileOG-db v5.1 [64].

### 4.7. Identification of Bacteriocin and Secondary Metabolite-Associated Genes

The identification of genes responsible for the production of ribosomally synthesized and post-translationally modified peptides (RiPPs) and other biosynthesis of secondary metabolites was performed using the antiSMASH web server [65]. Moreover, gene clusters related to bacteriocin synthesis were analyzed using the BAGEL4 web server [66].

### 4.8. Pan-Genome and Comparative Analysis

Pan-genome analysis was conducted on all 271 *E. lactis* genomes available on the NCBI database (accessed on 16 January 2024) compared with the *E. lactis* RB10 genome. The data were obtained by the Roary plugin [67], and the proteins with amino acid identities of 95% or higher were grouped into the same family. A maximum likelihood phylogenetic tree was constructed using FastTree v2.1 [68] and subsequently annotated and visualized using the Interactive Tree Of Life (iTOL) program v6 [69]. For comparative genomic analysis, genome sequences of the RB10 and its closely related genomes, including 50NA (Accession number: GCF_027925885.1), TATVAM-FAB81 (Accession number: GCF_046591675.1), E16 (Accession number: GCF_020556885.1), and 32-1 (Accession number: GCF_018397615.1) from the NCBI database, were compared and visualized using the jvenn webtool (https://jvenn.toulouse.inrae.fr/app/index.html, accessed on 16 May 2024).

### 4.9. Auto and Co-Aggregation Ability of RB10

The auto-aggregation ability of *E. lactis* RB10 was evaluated using a modified version of the previously described method [70]. An overnight culture of RB10 was harvested by centrifugation at 12,000× *g* for 15 min, washed twice with phosphate-buffered saline (PBS, pH 7.2), and resuspended in PBS to a final concentration of approximately 108 CFU/mL at 0 h (A_0_). The bacterial suspension was vortexed for 5 s and incubated at 37 °C, allowing sedimentation over time. Absorbance at 600 nm (OD600) of the upper suspension was measured at 2, 4, and 24 h (A_t_) to assess the aggregation. The auto-aggregation percentage was calculated using the formula:

Auto-aggregation (%) = [1 − (A_t_/A_0_)] × 100

where A_t_ represents suspension absorbance at different time points (1, 2, and 4 h) and A_0_ represents initial absorbance.

The co-aggregation ability of RB10 with foodborne pathogens was assessed by measuring optical density at OD600. Equal volumes of RB10 and the respective foodborne pathogens were mixed and incubated at 37 °C for 2, 4, and 24 h. The co-aggregation percentage was calculated using the formula:

Co-aggregation (%) = [1 − (A_Mix_/((A_RB10_ + A_P_)/2)] × 100

where A_RB10_ and A_P_ represent the absorbance of the RB10 suspension and the foodborne pathogen suspension alone, while A_Mix_ is the absorbance of the mixed solution.

All assays were conducted using three biological replicates, with each replicate performed in triplicate.

### 4.10. Antibacterial Activity Against Foodborne Pathogens

The antibacterial activity of *E. lactis* RB10 was assessed using the agar well diffusion method against foodborne pathogens, following a previously described method [71]. Overnight cultures of the foodborne pathogens were adjusted to a 0.5 McFarland standard in TSB and then spread onto Mueller-Hinton agar (MHA) plates. The cell-free supernatant (CFS) of RB10 was collected at 24 and 48 h by centrifugation at 8000× *g* for 10 min. One hundred microliters of each CFS sample were added to the wells and incubated at 37 °C for 16–18 h. The diameter of inhibition zones was measured to evaluate the antibacterial activity. The MRS broth was used as a control to ensure that the observed inhibition was not attributed to the culture medium itself. The experiment was conducted in three biological replicates, with each replicate performed in triplicate.

### 4.11. Statistical Analysis

All experimental results are presented as the mean values ± standard deviation (SD), derived from two or three independent experiments. Statistical analyses were conducted using one-way analysis of variance (ANOVA), followed by Tukey’s post hoc test to assess multiple comparisons. A *p*-value of <0.05 was considered statistically significant. All statistical procedures were performed using SPSS software (version 28.0.1.0; IBM Corp., Armonk, NY, USA).

## 5. Conclusions

In this study, the genomic and functional analyses of *E. lactis* RB10, isolated from goat feces, reveal its significant potential and antimicrobial capabilities. The strain exhibits antimicrobial activity against specific pathogens, supported by the presence of bacteriocin and secondary metabolite biosynthetic gene clusters, enabling it to compete with pathogens and survive in the gastrointestinal tract. While the strain exhibits a favorable safety profile, characterized by the absence of high-risk virulence factors and the presence of only intrinsic antimicrobial resistance genes, further functional validation and comprehensive safety studies are necessary before considering any application in food or clinical settings.

## Figures and Tables

**Table 1:** Antibiotic susceptibility profile of *E. lactis* RB10.

Antibiotics	Inhibition Zone (mm)	Interpretation
Ampicillin (10 μg)	19.00 ± 0.50	S
Chloramphenicol (30 μg)	21.92 ± 1.18	S
Clindamycin (2 μg)	7.83 ± 1.04	R
Erythromycin (15 μg)	11.00 ± 0.66	R
Gentamycin (10 μg)	9.42 ± 0.88	R
Kanamycin (30 μg)	0.00 ± 0.00	R
Streptomycin (10 μg)	0.00 ± 0.00	R
Tetracycline (30 μg)	28.58 ± 2.16	S
Vancomycin (30 μg)	21.92 ± 0.76	S

Note: S is susceptible, R is resistant. These classifications are determined based on the concentration breakpoints established by the CLSI guidelines.

**Table 2:** Genome features and information of *E. lactis* RB10.

Genome Features	RB10
Genome size (bp)	2,713,772
GC content (%)	38.3
Number of contigs	8
N50	1,148,270
L50	1
Number of CDSs	3375
Repeat region	1
tRNA	66
rRNA	12
tmRNA	1
Number of subsystems	233
Probability of being a human pathogen (%)	40
Classification	*Enterococcus lactis*
Closest placement reference	*E. lactis* (CCM 8412)
Average nucleotide identity (ANI)	98.06%

**Table 3:** Inhibition zones of *E. lactis* RB10’s CFS against foodborne pathogens. Data represent the mean ± SD of antimicrobial activity observed for each pathogen.

Pathogens	Inhibition Zone (mm)
*B. cereus* DMST 11098	12.33 ± 0.94
*E. coli* O157:H7	0.00 ± 0.00
*E. faecalis* DMST 4736	0.00 ± 0.00
*L. monocytogenes* DMST 17303	0.00 ± 0.00
*S.* Typhi DMST 22842	14.58 ± 0.12

## Data Availability

The genome of *E. lactis* RB10 has been submitted to the BioProject using the accession number PRJNA1261801 and BioSample with accession number SAMN48437941.

## Supplementary Materials

The following supporting information can be downloaded at: https://www.mdpi.com/article/10.3390/antibiotics14060612/s1, Table S1: Identification of virulence factor-related genes in *E. lactis* RB10 genome; Table S2: The identification of plasmids in the *E. lactis* RB10 genome; Table S3: Mobile genetic elements (MGEs) found in the *E. lactis* RB10 genome; Table S4: Unique genes identified in the RB10 genome.
