# Supplementary material for: Genomic Characterization and Safety Evaluation of Enterococcus lactis RB10 Isolated from Goat Feces
Source: Antibiotics (Basel). 2025 Jun 16;14(6):612. doi: 10.3390/antibiotics14060612 (PMC12189428; doi:10.3390/antibiotics14060612)
Supplement: Supplementary file 1 [file antibiotics-14-00612-s001.zip › antibiotics-3676674-supplementary.pdf]

Table S1. Identification of virulence factor-related genes in *E. lactis* RB10 genome.

Gene name	Product	Identity (%)	E-value
sgrA	Cell wall-anchored protein Sgra	96	0
cesC	ABC transporter ATP-binding protein	94	5.00E-05
ebpC	Endocarditis and biofilm-associated pilus major subunit Ebpc	83	2.00E-16
srtC	Sortase	89	9.00E-09
tufA	Elongation factor Tu	80	2.00E-06
fbpA	Fibronectin-binding protein	94	1.00E-04
srtC-2/srtC	Sortase	94	6.00E-04
carB	Carbamoyl phosphate synthase large subunit	86	0.002
bopD	Sugar-binding transcriptional regulator, LacI family	80	1.00E-71
scm	Collagen adhesin protein Scm	88	4.00E-62
tufA	Elongation factor Tu	83	1.00E-34
clpC	Endopeptidase Clp ATP-binding chain C	80	4.00E-19
lap	Listeria adhesion protein Lap	86	1.00E-16
efaA	Endocarditis specific antigen	83	9.00E-11
cap8D	Type 8 capsular polysaccharide synthesis protein Cap8D	87	6.00E-06
lpeA	Lipoprotein promoting cell invasion	87	6.00E-06
hasC	UTP--glucose-1-phosphate uridylyltransferase HasC	80	2.00E-05
clpE	ATP-dependent protease	100	2.00E-05
cpsA/uppS	Undecaprenyl diphosphate synthase	82	1.00E-11
cpsB/cdsA	Phosphatidate cytidylyltransferase	85	1.00E-08
kfiD	UDP-glucose 6-dehydrogenase	92	8.00E-07
hasB	UDP-glucose 6-dehydrogenase HasB	88	3.00E-06
acm	Collagen adhesin precursor Acm	96	0
clpP	ATP-dependent Clp protease proteolytic subunit	82	4.00E-33
gndA	NADP-dependent phosphogluconate dehydrogenase	81	2.00E-25
clpE	ATP-dependent protease	80	3.00E-21
EF0818	Polysaccharide lyase, family 8	94	1.00E-14
groEL	Chaperonin GroEL	94	3.00E-12
hasC	UTP--glucose-1-phosphate uridylyltransferase HasC	80	1.00E-05
clpC	Endopeptidase Clp ATP-binding chain C	87	4.00E-05
wbtM	Dtdp-D-glucose 4,6-dehydratase	94	2.00E-04
htpB	Hsp60, 60K heat shock protein HtpB	94	6.00E-04

Table S2. The identification of plasmids in the *E. lactis* RB10 genome.

Plasmid	Identity (%)	Position (bp)	Plasmid type	Accession number
rep1	97.59	141,815-142,766	repE(pKL0018)	AB290882
repUS15	98.18	92,977-94,018	repA(pNB2354p1)	CP004064

Table S3. Mobile genetic elements (MGEs) found in the *E. lactis* RB10 genome.

Gene Name	Major mobileOG category	Contig	Start (bp)	End (bp)
hsdM	Stability/transfer/defense	contig 2	26,065	27,660
hsdR	Stability/transfer/defense	contig 2	21,704	22,177
hsdR	Stability/transfer/defense	contig 2	22,138	24,693
insF	Integration/excision	contig 2	45,216	46,052
insF	Integration/excision	contig 2	62,931	63,194
insF	Integration/excision	contig 2	63,218	63,766
insF	Integration/excision	contig 2	149,866	150,636
int	Integration/excision	contig 2	29,044	29,358
relE	Stability/transfer/defense	contig 2	68,696	68,911
repS	Replication/recombination/repair	contig 2	141,822	142,415
ssb	Replication/recombination/repair	contig 2	131,300	131,785
tnp	Integration/excision	contig 2	17,008	17,466
tnp	Integration/excision	contig 2	20,933	21,613
tnp	Integration/excision	contig 2	35,204	36,112
tnp	Integration/excision	contig 2	43,216	43,674
tnp	Integration/excision	contig 2	44,046	44,954
tnp	Integration/excision	contig 2	46,088	46,378
tnp	Integration/excision	contig 2	63,917	64,093
tnp	Integration/excision	contig 2	81,262	81,621
tnp	Integration/excision	contig 2	87,102	87,785
tnp	Integration/excision	contig 2	89,029	89,718
tnp	Integration/excision	contig 2	90,451	91,359
tnp	Integration/excision	contig 2	137,084	137,575
tnp	Integration/excision	contig 2	149,540	149,830
tnpA	Integration/excision	contig 2	5,196	5,630
tnpA	Integration/excision	contig 2	14,244	14,678
tnpA	Integration/excision	contig 2	16,786	16,971
topB	Transfer	contig 2	127,343	129,049
topB	Transfer	contig 2	129,046	129,543
topB	Transfer	contig 2	143,172	144,005
topB	Transfer	contig 2	144,051	144,560
topB	Transfer	contig 2	144,640	144,975
tra905	Integration/excision	contig 2	4,666	5,142
tra905	Integration/excision	contig 2	52,346	53,287
tra905	Integration/excision	contig 2	77,922	78,773
tra905	Integration/excision	contig 2	139,150	140,091
cca	Phage	contig 3	374,385	375,593

gyrB	Replication/recombination/repair	contig 3	344,917	345,792
gyrB	Replication/recombination/repair	contig 3	346,139	346,906
hup	Replication/recombination/repair	contig 3	380,263	380,538
lexA	Phage	contig 3	179,809	180,228
parC	Replication/recombination/repair	contig 3	342,559	343,437
parC	Replication/recombination/repair	contig 3	343,434	344,852
recJ	Replication/recombination/repair	contig 3	181,371	183,668
recQ	Replication/recombination/repair	contig 3	254,855	256,648
recU	Replication/recombination/repair	contig 3	165,615	166,262
rlmL	Stability/transfer/defense	contig 3	162,789	163,238
rlmL	Stability/transfer/defense	contig 3	163,313	163,957
rnhB	Replication/recombination/repair	contig 3	357,730	358,497
rph	Replication/recombination/repair	contig 3	198,133	199,251
smf	Replication/recombination/repair	contig 3	356,818	357,348
smf	Replication/recombination/repair	contig 3	357,405	357,674
thyA	Phage	contig 3	366,333	367,280
topA	Replication/recombination/repair	contig 3	354,618	356,750
topB	Transfer	contig 3	105,623	105,871
topB	Transfer	contig 3	106,032	107,801
xerC	Integration/excision	contig 3	352,264	353,076
xerD	Integration/excision	contig 3	395,631	396,518
xlyA	Phage	contig 3	225,674	226,300
xlyA	Phage	contig 3	322,079	322,705
cas2	Stability/transfer/defense	contig 4	591,819	592,136
cinA	Transfer	contig 4	138,056	139,297
clpB	Phage	contig 4	243,368	245,596
clpB	Phage	contig 4	245,593	245,853
dnaA	Replication/recombination/repair	contig 4	284,792	285,517
dnaA	Replication/recombination/repair	contig 4	285,487	286,125
dnaB	Replication/recombination/repair	contig 4	297,669	298,166
dnaB	Replication/recombination/repair	contig 4	298,253	299,035
dnaN	Replication/recombination/repair	contig 4	286,322	287,452
dut	Transfer	contig 4	154,837	155,118
dut	Transfer	contig 4	155,154	155,363
exoA_2	Replication/recombination/repair	contig 4	931,273	932,082
ftsH	Phage	contig 4	84,433	86,544
gyrA	Replication/recombination/repair	contig 4	290,993	293,464
gyrB	Replication/recombination/repair	contig 4	289,028	290,665
gyrB	Replication/recombination/repair	contig 4	290,605	290,973
hsdM	Stability/transfer/defense	contig 4	268,859	270,454

hsdR	Stability/transfer/defense	contig 4	264,499	267,648
insK	Integration/excision	contig 4	439,553	440,080
int	Integration/excision	contig 4	271,881	272,840
int	Integration/excision	contig 4	936,075	937,034
mutL	Replication/recombination/repair	contig 4	428,183	429,232
mutL	Replication/recombination/repair	contig 4	429,183	430,292
mutS	Replication/recombination/repair	contig 4	425,520	428,165
mutS2	Replication/recombination/repair	contig 4	999,429	1,001,789
ndoA_1	Stability/transfer/defense	contig 4	482,348	482,665
nfo	Replication/recombination/repair	contig 4	1,098,259	1,099,161
oppB	Transfer	contig 4	387,480	388,442
oppC	Transfer	contig 4	388,469	389,383
oppD	Transfer	contig 4	317,073	318,002
oppD	Transfer	contig 4	385,531	386,241
oppD	Transfer	contig 4	386,219	386,536
oppF	Transfer	contig 4	221,344	222,150
oppF	Transfer	contig 4	386,537	387,025
oppF	Transfer	contig 4	387,064	387,480
parB	Replication/recombination/repair	contig 4	261,198	261,752
parB	Replication/recombination/repair	contig 4	261,803	262,087
radA	Replication/recombination/repair	contig 4	153,707	154,774
recA	Replication/recombination/repair	contig 4	136,898	137,947
recF	Replication/recombination/repair	contig 4	287,908	288,462
recF	Replication/recombination/repair	contig 4	288,447	289,031
recG	Replication/recombination/repair	contig 4	381,817	382,119
recG	Replication/recombination/repair	contig 4	382,194	383,852
rex	Stability/transfer/defense	contig 4	967,452	968,015
rnpA	Replication/recombination/repair	contig 4	283,854	284,018
ruvA	Replication/recombination/repair	contig 4	434,572	435,174
ruvB	Replication/recombination/repair	contig 4	435,187	436,188
soj_2	Replication/recombination/repair	contig 4	262,074	262,841
ssb	Replication/recombination/repair	contig 4	294,041	294,562
tnp	Integration/excision	contig 4	349,582	350,490
tnp	Integration/excision	contig 4	543,915	544,676
topB	Replication/recombination/repair	contig 4	833,745	835,079
topB	Replication/recombination/repair	contig 4	835,019	835,660
topB	Replication/recombination/repair	contig 4	835,657	835,761
tra905	Integration/excision	contig 4	545,668	546,288
tra905	Integration/excision	contig 4	546,541	546,837
ung	Replication/recombination/repair	contig 4	927,066	927,746

uvrC	Replication/recombination/repair	contig 4	1,002,372	1,003,622
uvrC	Replication/recombination/repair	contig 4	1,003,565	1,004,149
xlyA	Phage	contig 4	770,160	770,768
nusA	Phage	contig 6	138,612	139,787
polC	Replication/recombination/repair	contig 6	133,623	134,804
polC	Replication/recombination/repair	contig 6	134,801	136,897
polC	Replication/recombination/repair	contig 6	136,894	137,973
tag	Replication/recombination/repair	contig 6	56,961	57,533
clpB	Phage	contig 7	42,732	45,341
clpX	Phage	contig 7	122,001	123,251
dnaI	Replication/recombination/repair	contig 7	51,765	52,511
dnaJ	Replication/recombination/repair	contig 7	165,632	166,798
dnaK	Phage	contig 7	166,949	168,778
g34	Phage	contig 7	18,667	18,915
g34	Phage	contig 7	18,933	19,793
gp59	Phage	contig 7	5,583	5,780
ligA	Replication/recombination/repair	contig 7	109,542	111,578
mutM	Replication/recombination/repair	contig 7	55,877	56,713
pcrA	Replication/recombination/repair	contig 7	111,595	112,734
pcrA	Replication/recombination/repair	contig 7	112,734	113,744
polA	Replication/recombination/repair	contig 7	56,758	57,609
polA	Replication/recombination/repair	contig 7	57,714	58,292
polA	Replication/recombination/repair	contig 7	58,348	59,400
relE	Stability/transfer/defense	contig 7	172,423	172,638
terL	Phage	contig 7	23,154	23,648
terL	Phage	contig 7	23,703	24,443
terS	Phage	contig 7	24,427	25,044
uvrA	Replication/recombination/repair	contig 7	147,691	148,593
uvrA	Replication/recombination/repair	contig 7	148,644	149,876
uvrA	Replication/recombination/repair	contig 7	149,942	150,505
uvrB	Replication/recombination/repair	contig 7	150,595	152,508
yqaJ	Integration/excision	contig 7	31,881	32,201
yqaJ	Integration/excision	contig 7	32,198	32,620
yqaK	Replication/recombination/repair	contig 7	30,697	31,587
clpB	Phage	contig 8	489,871	492,105
clpP	Phage	contig 8	311,387	311,980
copR	Transfer	contig 8	541,098	541,598
copR	Transfer	contig 8	541,598	541,783
dinB	Replication/recombination/repair	contig 8	343,790	344,698
dnaE	Replication/recombination/repair	contig 8	531,089	533,968

dnaE	Replication/recombination/repair	contig 8	533,968	534,399
dnaX	Replication/recombination/repair	contig 8	336,627	337,430
dnaX	Replication/recombination/repair	contig 8	337,391	338,371
ftsK	Replication/recombination/repair	contig 8	202,691	205,138
ftsZ	Replication/recombination/repair	contig 8	396,541	397,569
groL	Phage	contig 8	121,037	122,662
groS	Phage	contig 8	120,701	120,985
holB	Replication/recombination/repair	contig 8	340,393	341,331
mecA	Transfer	contig 8	49,454	50,107
mutY	Replication/recombination/repair	contig 8	481,638	482,816
nusB	Phage	contig 8	377,468	377,917
nusG	Phage	contig 8	67,816	68,361
oppD	Transfer	contig 8	212,379	212,777
oppD	Transfer	contig 8	212,867	213,448
oppF	Transfer	contig 8	213,611	214,390
prgZ	Transfer	contig 8	208,228	209,889
radC	Replication/recombination/repair	contig 8	303,308	303,640
radC	Replication/recombination/repair	contig 8	303,649	303,984
rarA	Replication/recombination/repair	contig 8	21,379	22,401
recD2	Transfer	contig 8	61,644	62,708
recD2	Transfer	contig 8	62,737	63,471
recD2	Transfer	contig 8	63,528	64,211
recR	Replication/recombination/repair	contig 8	338,731	339,327
rex	Stability/transfer/defense	contig 8	297,405	298,052
rnj	Replication/recombination/repair	contig 8	304,591	304,788
rnj	Replication/recombination/repair	contig 8	304,822	306,267
tmk	Phage	contig 8	339,391	340,035
tnpB	Integration/excision	contig 8	214,402	214,590
tnpB	Integration/excision	contig 8	214,701	214,952
tnpB	Integration/excision	contig 8	214,949	215,410
xseA	Replication/recombination/repair	contig 8	378,867	379,337
xseA	Replication/recombination/repair	contig 8	379,438	380,226

Table S4. Unique genes identified in the RB10 genome.

Gene	Description
acr3	Arsenical-resistance protein Acr3
amiA	Oligopeptide-binding Sprotein AmiA
arsA	Arsenical pump-driving ATPase
arsD	Arsenical resistance operon trans-acting repressor ArsD
bmrA	Multidrug resistance ABC transporter ATP-binding/permease protein BmrA
cas9	CRISPR-associated endonuclease Cas9
cutD	Choline trimethylamine-lyase activating enzyme
dapE	putative succinyl-diaminopimelate desuccinylase
dnaA	Chromosomal replication initiator protein DnaA
dprA	DNA processing protein DprA
ecsA	ABC-type transporter ATP-binding protein EcsA
essB	Type VII secretion system protein EssB
essC	Type VII secretion system protein EssC
fhs2	Formate--tetrahydrofolate ligase 2
folD	Bifunctional protein FolD protein
gatA	Glutamyl-tRNA(Gln) amidotransferase subunit A
glyQ	Glycine--tRNA ligase alpha subunit
gpsA	Glycerol-3-phosphate dehydrogenase [NAD(P)+]
guaA	GMP synthase [glutamine-hydrolyzing]
gyrB	DNA gyrase subunit B
hrcA	Heat-inducible transcription repressor HrcA
lacD	Tagatose 1%2C6-diphosphate aldolase
manR	Transcriptional regulator ManR
merA	Mercuric reductase
merR	Mercuric resistance operon regulatory protein
metB	Cystathionine gamma-synthase
metG	Methionine--tRNA ligase
mgtE	Magnesium transporter MgtE
mnmA	tRNA-specific 2-thiouridylase MnmA
murG	UDP-N-acetylglucosamine--N-acetylmuramyl-(pentapeptide) pyrophosphoryl-undecaprenol N-acetylglucosamine transferase
nagR	HTH-type transcriptional repressor NagR
nrdR	Transcriptional repressor NrdR
ntpJ	Potassium/sodium uptake protein NtpJ
parE	DNA topoisomerase 4 subunit B
pdhD	Dihydrolipoyl dehydrogenase
pdp	Pyrimidine-nucleoside phosphorylase
pepDA	Dipeptidase A

phoU	Phosphate-specific transport system accessory protein PhoU
pnp	Polyribonucleotide nucleotidyltransferase
prfA	Peptide chain release factor 1
proA	Gamma-glutamyl phosphate reductase
proS	Proline--tRNA ligase
purC	Phosphoribosylaminoimidazole-succinocarboxamide synthase
purH	Bifunctional purine biosynthesis protein PurH
purL	Phosphoribosylformylglycinamidine synthase subunit PurL
pyrDA	Dihydroorotate dehydrogenase A (fumarate)
queH	Epoxyqueuosine reductase QueH
recN	DNA repair protein RecN
rexB	ATP-dependent helicase/deoxyribonuclease subunit B
rhaD	Rhamnulose-1-phosphate aldolase
rny	Ribonuclease Y
sdpR	Transcriptional repressor SdpR
tkt	Transketolase
xerS	Tyrosine recombinase XerS
xseA	Exodeoxyribonuclease 7 large subunit
yadG	Putative ABC transporter ATP-binding protein YadG
yhaN	Putative protein YhaN
